# Antagonism between salicylate and the cAMP signal controls yeast cell survival and growth recovery from quiescence

**DOI:** 10.15698/mic2018.07.640

**Published:** 2018-03-26

**Authors:** Maurizio D. Baroni, Sonia Colombo, Enzo Martegani

**Affiliations:** 1 Dipartimento di Biologia, Università di Padova, 35133 Padova, Italy.; 2 Dipartimento di Biotecnologie e Bioscienze, Università Milano Bicocca, 20126 Milano, Italy.

**Keywords:** Salicylate, cyclic AMP, cellular growth, cell death, yeast, NSAIDs

## Abstract

Aspirin and its main metabolite salicylate are promising molecules in preventing cancer and metabolic diseases. *S. cerevisiae* cells have been used to study some of their effects: (i) salicylate induces the reversible inhibition of both glucose transport and the biosyntheses of glucose-derived sugar phosphates, (ii) Aspirin/salicylate causes apoptosis associated with superoxide radical accumulation or early cell necrosis in MnSOD-deficient cells growing in ethanol or in glucose, respectively. So, treatment with (acetyl)-salicylic acid can alter the yeast metabolism and is associated with cell death. We describe here the dramatic effects of salicylate on cellular control of the exit from a quiescence state. The growth recovery of long-term stationary phase cells was strongly inhibited in the presence of salicylate, to a degree proportional to the drug concentration. At high salicylate concentration, growth reactivation was completely repressed and associated with a dramatic loss of cell viability. Strikingly, both of these phenotypes were fully suppressed by increasing the cAMP signal without any variation of the exponential growth rate. Upon nutrient exhaustion, salicylate induced a premature lethal cell cycle arrest in the budded-G2/M phase that cannot be suppressed by PKA activation. We discuss how the dramatic antagonism between cAMP and salicylate could be conserved and impinge common targets in yeast and humans. Targeting quiescence of cancer cells with stem-like properties and their growth recovery from dormancy are major challenges in cancer therapy. If mechanisms underlying cAMP-salicylate antagonism will be defined in our model, this might have significant therapeutic implications.

## INTRODUCTION

The nonsteroidal anti-inflammatory drugs (NSAIDs) can act through multiple mechanisms, some of which are also driven by their metabolites. This is the case of Aspirin (acetylsalicylic acid), one of the most used internationally drugs due to its anti-inflammatory, anti-thrombotic, anti-pyretic and analgesic properties 1, and its main metabolite salicylate (2-hydroxybenzoic or salicylic acid, SA). Natural salicylates are among the oldest drugs used by humans since they are ubiquitous in plants (where they exert important functions) 2 and particularly abundant in some species (e.g. in the willow *Salix alba*, where they were originally extracted from its bark) 3.

Aspirin is a synthetic mono-acetylated SA derivative that can transfer *in vivo *its acetyl group to eight different amino acids of hundreds of proteins involved in key biological and cellular functions with widespread but, in most cases, still largely unknown consequences (4 and studies discussed therein). In this context, the acetylation of cyclooxygenases (COX1 and 2) contributes to varying degrees and with different mechanisms to the Aspirin multiple effects 1,5.

Aspirin has a very short half-life in the body (∼15-20 minutes) due to its rapid hydrolysis 6, both spontaneous and via enzyme-catalyzed *in vivo* hydrolysis. In particular, it is rapidly broken down to salicylate by both serum and cellular esterases so that only a small fraction can reach the peripheral tissues 7. In addition, unlike platelets the nucleated cells are able to resynthesize or deacetylate its acetylated targets. As a consequence, Aspirin must also be considered a pro-drug, which is quickly transformed into its main active metabolite salicylate 3. This latter is much more stable having a half-life ranging between 3-5 hours (in most cases) but half-lives of 30-40 hours has been recorded (its dosage and physiopathological factors markedly influencing the pathways and rate of metabolism) 8. The peak serum concentrations of SA, following oral Aspirin administration in both laboratory animals and humans, are also much higher than those of Aspirin 8,9. Finally, salicylic acid is obtained from dietary intake, with higher levels of SA in vegetarians overlapping with levels in patients on low-dose Aspirin regimens 10.

Daily low-dose Aspirin taken for cardioprevention has been also causally linked to a decreased incidence of both gastrointestinal carcinomas and (less strongly) some other cancers. There are plausible COX-dependent as well as many COX-independent multiple mechanisms underlying the cancer preventive efficacy of Aspirin/SA. These involve several Aspirin/SA molecular targets that appear to act by decreasing inflammation, platelet activation, glucose metabolism, mitochondrial oxidative phosphorylation, protein translation and cell proliferation as well as by enhancing apoptosis, differentiation, stress responses, tumour immunosurveillance and autophagy (summarized and discussed in 11). Most of these cell processes are conserved among eukaryotes.

The elucidation of the anticancer mechanisms of Aspirin/salicylate can greatly benefit from the use of experimental models, including *Saccharomyces cerevisiae* as shown by some previous pioneering studies in budding yeast 12. These studies strongly indicate that at least some of the above mentioned cell processes are similarly regulated by Aspirin/SA in *S. cerevisiae* cells. Briefly, the treatment of yeast cells with Aspirin and/or salicylic acid can reversibly repress the yeast glucose transport and metabolism and it is associated with programmed cell death (PCD) (discussed in 12). Previous studies have indicated SA stereospecific binding sites located within yeast cells and SA reversible inhibition of glucose transport 13 and inhibition of uptake and distribution of ^14^C from [^14^C]glucose into sugar phosphates, uridine diphosphoglucose and, more markedly, trehalose 6-phosphate (T6P) and trehalose 14. In addition, studies on the growth inhibitory and proapoptotic effects of Aspirin and the derived salicylate in *S. cerevisiae* indicated that yeast mitochondria constitute one of its critical targets (reviewed in 12). Among factors which play roles in PCD induced by Aspirin/SA are ROS (reactive oxygen species) and mitochondrial dysfunctions with inhibition of the electron transport chain and aerobic respiration. In addition, Aspirin/SA induced apoptosis is associated with superoxide radical accumulation and NAD(P)H oxidation 15, and low doses of salicylate can confer long-term cytoprotective resistance against H_2_O_2_-induced oxidative stress 16. This Aspirin/SA PCD model also includes decrease of ΔΨ_m_, release of CytC (cytochrome c) and pH lowering 17 but it was limited to a condition of ethanol metabolism and MnSOD (manganese dependent superoxide dismutase)deficiency. In contrast, early cell necrosis has been observed for a glucose cultured population of yeast cells treated with Aspirin 18. Finally, a dominant negative effect of salicylate on yeast heat-shock induced transcription linked to intracellular pH decrease has also been described 19. Hence, although still limited this picture seems to be consistent to that observed in mammalian cells. Noteworthy, yeast cells have been successfully used to further deepen our knowledge on other NSAIDs, such as Diclofenac (and related drugs) 20 and Ibuprofen 21.

The present study gives a further significant contribution to the field by describing totally new phenotypes of yeast cells treated with salicylate. We show how salicylate can strongly inhibit exit from G0 phase by delaying or even blocking growth reactivation of profoundly quiescent cells. We also describe a dramatic antagonism between salicylate and cAMP (a ubiquitous signal known to regulate growth/proliferation and other vital processes 22), which determines the ability of cells to resume growth and their own survival. We also argue that salicylate could interact not only with the cAMP pathway but with other metabolic hubs too. Preliminary data showing that salicylate also impairs cell cycle arrest in G1/GO upon nutrient exhaustion further support this view. Mechanisms allowing the interplay between salicylate and PKA may be evolutionarily conserved and their knowledge can have future therapeutic implications in prevention of cancer and metabolic diseases.

## RESULTS

### Salicylate has a modest and transient effect on cellular growth of log phase cells

**Figure 1:**
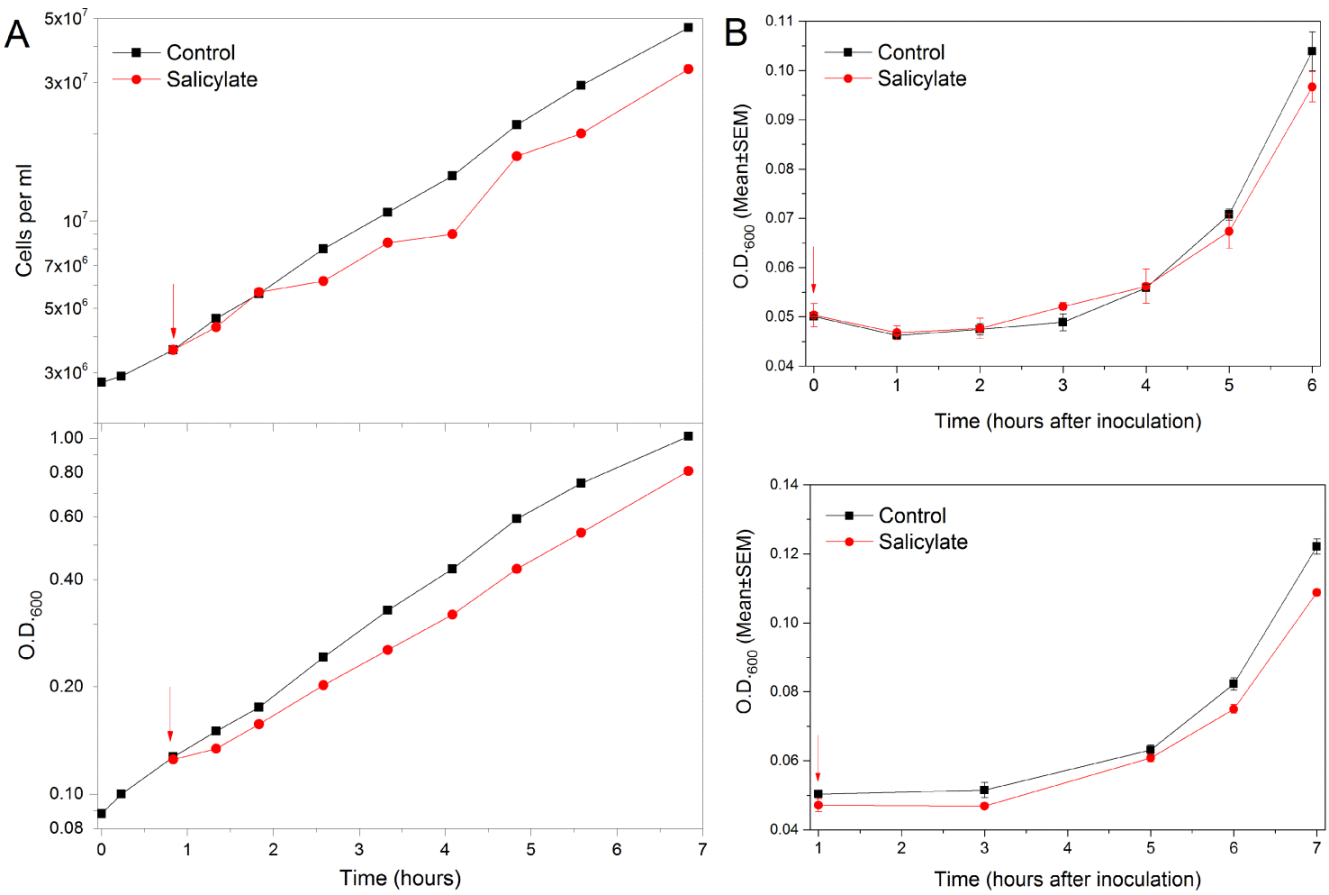
FIGURE 1: Limited salicylate effects on proliferation of active yeast populations. **(A) **Mild transient inhibition of yeast exponential growth with salicylate. An exponentially growing culture of wt yeast cells (black) was supplied (red arrow) with 3 mM salicylate (red). The cell number per ml (upper graph) and the Optical Density (OD) at 600nm (lower graph) were monitored over time. The Log_10_ of each value was graphically plotted versus time. After a transient and modest growth inhibition due the drug the culture Doubling Time (DT) returned similar to that of untreated control. DTs from the cell number increases: 1.66 (control) and 1.69 (SA) hours. DTs from OD_600_ values: 2.1 (control) and 2.2 (SA) hours. DTs were calculated in late log phase (last 5 points), so are expected to be longer if derived from O.D._600_. **(B) **Unaltered *lag phase* before growth recovery of chronologically young yeast populations supplied with salicylate. wt yeast cells were re-inoculated into fresh medium after 2 days (*upper panel*) or 4 days (*lower panel*) spent into stationary phase. OD_600nm_ values were monitored in control untreated cells (black) and cells supplied with 3 mM salicylate (red). Values of Means (±SEM) derived from three independent repeats (each performed in triplicate) were plotted against time.

An exponentially growing culture of wt (wild type) yeast cells (W303-1A) was treated with 3 mM salicylate and both the cell number and the of OD_600_ values were followed over time. Salicylate only induced a transient and limited inhibition of both the cell proliferation (followed as cell number increase) and biomass accumulation (monitored as OD increase) (Figure 1A). After less than two hours from its addition the culture regained a growth rate comparable to that of the untreated control one (Figure 1A and Supplementary Figures S1A, C). The percent of budded or S/G2/M cells (Supplementary Figures S1A, B) and the cell volume distribution (data not shown) were also unaltered by the drug during the exponential phase of growth.

### Salicylate impairs the G1/G0 arrest of yeast cells approaching the stationary phase

Interestingly, salicylate supplied during log-phase impaired the cell’s ability to properly arrest in G1 phase upon nutrient exhaustion. Indeed, the drug caused a large fraction of cells approaching the stationary phase to prematurely arrest cell division in the budded-G2/M phase (Supplementary Figures S2B, D). Overall biomass accumulation was also inhibited as indicated by both the smaller OD_600_ values in the SA-treated culture and the reduction of cell volumes (Supplementary Figures S2A-E). In addition, the presence of very small budded cells in the stationary phase suggested there might be an altered cell size control (Supplementary Figures S2A, B). Virtually all starved salicylate treated cells became unviable (Supplementary Figure 2B) (the volume reduction was also partially due to cell shrinkage). All the phenotypic traits were independent of the genetic background and were scarcely influenced by better nutritional conditions (Supplementary Figures S2A-C). Surprisingly, at least in solid media supplemented with 3 mM SA cells were able to accumulate glycogen, though to a lesser extent than control cells (data not shown). Glycogen is a storage carbohydrate marking the G0-quiescent phase 22. So, the massive loss of viability seen in liquid media might be due more specifically to a defective cell cycle arrest rather than a G0 defect. Alternatively, the upper cells of the colonies might come into contact with much less SA respect to the medium concentration. Different quiescence parameters should be appropriately probed to establish the cause of cell death.

**Figure 2:**
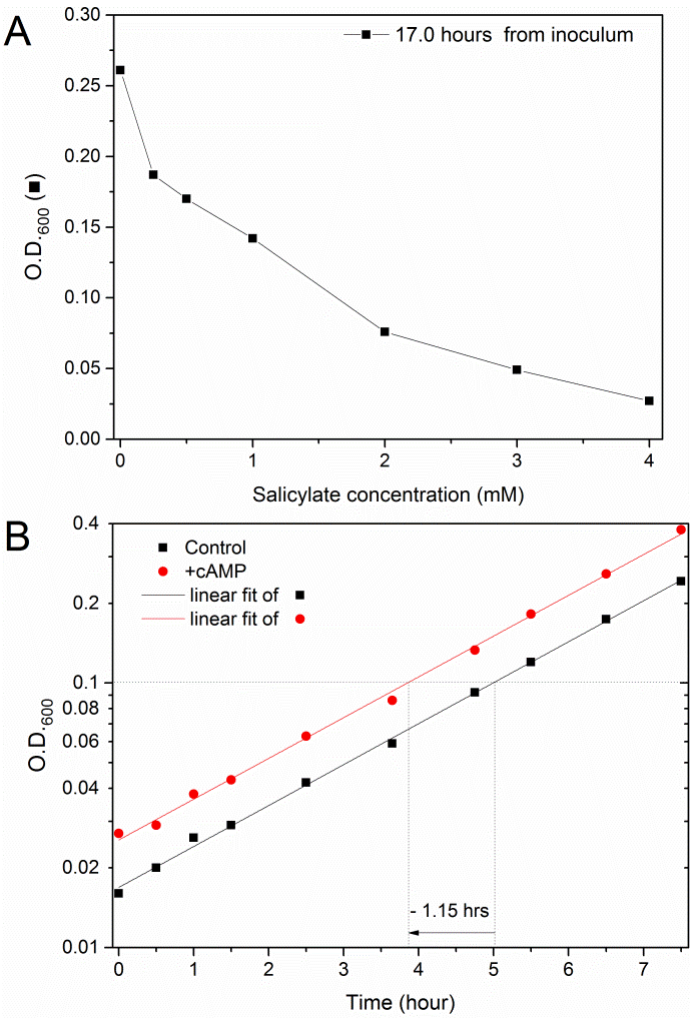
FIGURE 2: Opposite effects of salicylate and cAMP signalling pathway on yeast growth reactivation. **(A) **Growth reactivation delay of chronologically aged yeast cells induced by salicylate. wt cells arrested into stationary phase for 22 days were inoculated into fresh medium in the presence of different concentrations of salicylic acid. The cellular density was measured as OD_600nm _after 17 hours (black) obtaining the salicylate dose-response curves shown in figure. **(B) **Early growth reactivation elicited by cAMP in chronologically aged yeast cells. A 18 days-aged *cyr1*Δ*pde2*Δ*msn2*Δ*msn4*Δ mutant strain was inoculated into fresh medium supplied (red) or not (black square) with 3 mM cAMP. The Log_10_ of OD_600nm _values monitored during mid-log phase were plotted versus time (hours) and fitted with linear regression curves. As the curves were parallel, the time difference at a same mid-log cell density is a convenient estimation of the difference between *lag times* before growth in the two conditions. cAMP pushed cells to reactivate growth ∼1.15 hours earlier than control cells, as shown graphically.

### The *lag phase* duration after refeeding short-term stationary phase cultures is unaffected by salicylate

We then tested the ability of cells to restart growing in the presence of salicylate after a short period spent in a quiescence state. Pilot experiments suggested that after a few days into stationary phase the reactivation of growth was not affected by 3 mM salicylate (data not shown). Indeed, the *lag time* before growth was virtually unaffected by the drug until ∼4 days of dormancy (Figure 1B and Supplementary Figure S3B). In contrast, its duration was sensitive to the time the cells previously spent in G0, as expected (Supplementary Figures S3A, B). The growth rate during the early exponential phase was also unaltered or poorly affected by salicylate (Figures 1B and S3B).

### Salicylate strongly inhibits the growth recovery of chronologically aged yeast cells

*S. cerevisiae *is one of the most used models to investigate the molecular processes of aging in eukaryotes. Budding yeast cells deprived of essential nutrients undergo a process of chronological aging that induces profound metabolic changes 23. As salicylate appears to affect sugar metabolism/sensing in both yeast and higher eukaryotes (more details in *Introduction* and *Discussion*) we repeated the test by using long-term (∼3 weeks) stationary phase wt populations. The cell densities monitored after many hours in fresh medium containing various SA concentrations showed that the growth recovery of these aged cells can be dramatically impaired by salicylate, in proportion to the drug concentration (Figure 2A). The effect is evident even at the lowest used concentration (250 μM; see also Supplementary Figures S4A, B and Bonferroni statistical analyses in Figure S4C, left panel), which is similar to blood SA concentrations reached during therapies with Aspirin (going from a minimum of ∼0.02-0.1 mM to a maximum of ∼1-2 mM concentration) 8,9. The drug effects appear to be reversible (preliminary data in Supplementary Figure S4D) in agreement with previous reports 12,13.

### PKA activation induces an early reactivation of growth and suppresses the delay in growth recovery induced by salicylate

In budding yeast, cyclic AMP (cAMP) is produced in response to sensing and catabolism of fermentable carbon sources (in particular glucose) and several other nutrients. cAMP indeed is a key signal for driving cells out of a quiescent state 22. So, in principle the activation of cAMP-dependent kinases (PKAs) might counteract the growth inhibition due to salicylate.

The quadruple *cyr1*Δ*pde2*Δ*msn2*Δ*msn4*Δ mutant strain (GG104, isogenic to W303-1A), missing the essential adenylate cyclase encoding gene (*CYR1 or CDC35*), is unable to produce cAMP but it shows a good viability due to the inactivation of the main stress transcription factors Msn2p and Msn4p, two powerful PKA antagonists 24. In the same mutant cells it is possible to conveniently control the level of PKA activation by simply adding different concentrations of cAMP into the culture medium (this is greatly favoured by the inactivation of the high-affinity cAMP phosphodiesterase Pde2p 25,26).

**Figure 3:**
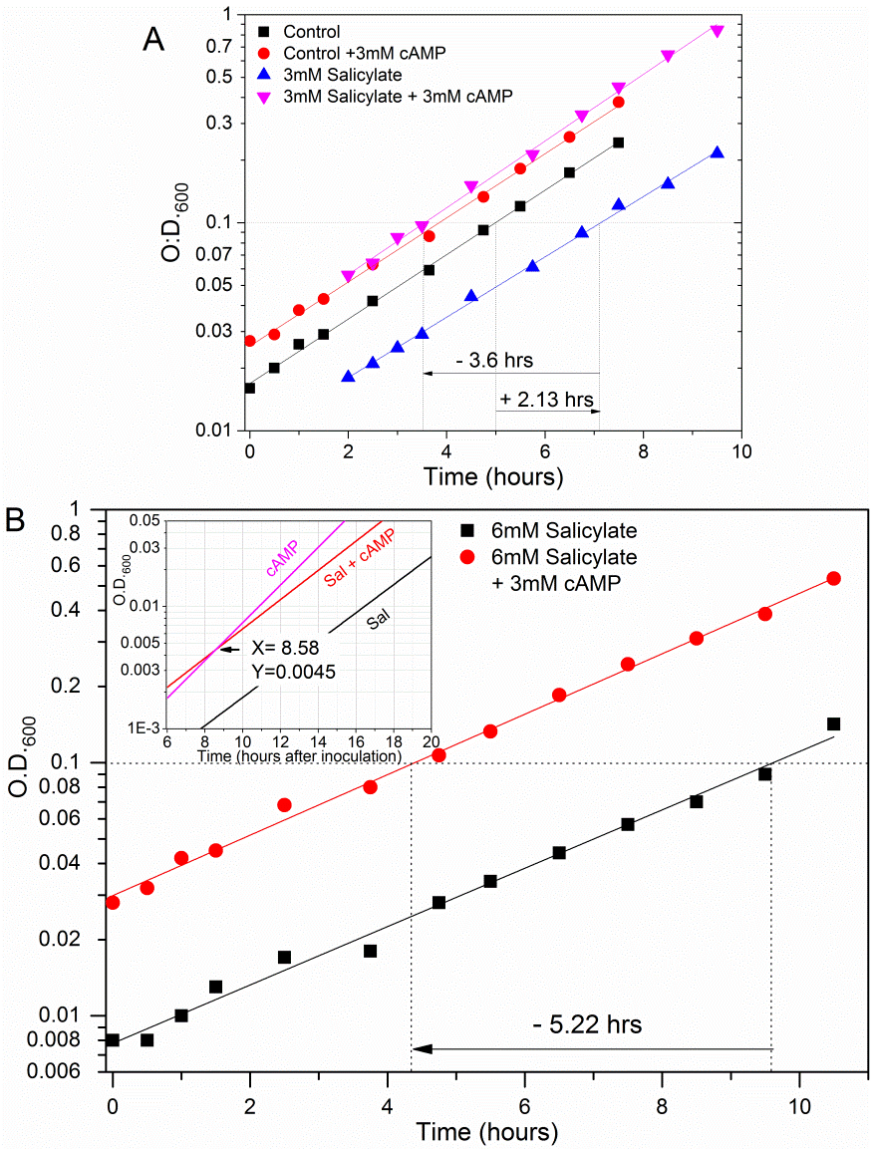
FIGURE 3: Specificity and efficacy of PKA suppressive activity. **(A) **Dominant cAMP effect in suppressing the SA-induced delay of cell growth recovery. *cyr1*Δ*pde2*Δ*msn2*Δ*msn4*Δ long-term quiescent cell populations were supplied (red) or not (black) with 3 mM cAMP or with 3 mM salicylate plus (pink) or minus (blue) 3 mM cAMP when inoculated into fresh medium, monitoring growth by measuring OD at 600nm. The Log_10_ values of the experimental points were plotted against time (hours) and fitted with linear regression curves. The variations of the time in which growth is reactivated in the different conditions are estimated as explained in the previous figure legend. 3 mM salicylate caused a delay in growth recovery of ∼2.13 hours compared to the untreated control (represented by a right pointing arrow) whereas the culture experiencing both cAMP and salicylate could start the growing phase ∼3.6 hour earlier than that with the drug alone (left pointing arrow). Noteworthy, its growth curve overlapped that of cells treated only with cAMP. **(B) **Specificity of cAMP effects for the reactivation but not the rate of cell growth. *cyr1*Δ*pde2*Δ*msn2*Δ*msn4*Δ mutant cells were treated with 6 mM salicylate and supplied (red) or not (black) with 3 mM cAMP as described in previous figure. The Log_10_ of OD_600nm_ values taken during the time course experiment were fitted with linear regression curves. At this dosage the drug increased the log-phase generation time from ∼2 to 2.61 hours irrespective of the cAMP presence. However, the addition of cAMP still shifted much earlier, namely ∼5.22 hours (left pointing arrow), the growth recovery of the culture. (The figure is representative of the results of 5 independent experiments). **Inset. **The regression curves of the above graph are drawn (line colours as in the main graph) together with that of a mutant cell culture supplied with cAMP only (lilac line). The coordinates of the crossing point (arrow) between the two cultures with cAMP are indicated. In particular, the "6 mM SA+cAMP" and "cAMP only" curves converged at about the cell density of inoculums, namely OD_600_≅0.005, and at a time of ∼8.6 hours (as determined with a graphing software; see *Materials and Methods*). Note that the moment of inoculation is put here as time zero.

We first established that cAMP signal and, perhaps more surprisingly, the activity of Msn2p and Msn4p are not needed for the salicylate induced phenotypes. Indeed, the *cyr1*Δ*pde2*Δ*msn2*Δ*msn4*Δ mutant challenged with salicylate showed both a defective G1/G0 arrest and small cell size upon starvation (Supplementary Figure S2C and data not shown) and growth recovery inhibition (Figure 3, fully described below). On the other side, aged (18 days) *cyr1*Δ*pde2*Δ*msn2*Δ*msn4*Δ cells re-inoculated into a fresh medium underwent a faster activation of growth (exit from G0) if supplied with cAMP. The effect was specific as the cyclic nucleotide did not alter the steady state growth rate during the mid-log phase (Figure 2B); this fact also allowed us to conveniently quantify the advancement of G0 exit driven with cAMP by measuring the time difference between the curves at a same cell density. In particular, 3 mM cAMP pushed cells to reactivate growth (1.15 hours earlier than controls. These experiments fully confirmed the role of cAMP as a limiting signal for exit from G0 and growth reactivation.

We next asked if salicylate and PKAs can exert a truly antagonistic effect on the same cells. Strikingly, when quiescent GG104 cells were treated with 3 mM cAMP they behaved irrespective of the presence of 3 mM salicylate, a concentration that instead per se produced a growth recovery delay of ∼2.13 hours compared to the untreated control. Indeed, the culture experiencing both cAMP and salicylate could start the growth phase ∼3.6 hour earlier than that with only salicylate, behaving just like cells supplied with cAMP alone (Figure 3A and Supplementary Figure S6A). So, the stimulatory effect of cAMP appeared to be fully dominant and it was roughly proportional to the concentration of the signal molecule (Supplementary Figure S6B). The 3 mM exogenous cAMP concentration is likely saturating. As already noted the exponential phase growth rate was largely unaffected by both cAMP and 3 mM salicylate (Figures 2B, 3A and S6A, B; Doubling Time (DT) was ∼2.0 hours for all cultures).

### cAMP cannot suppress the decrease of exponential growth rate due to a high salicylate dosage 

When cells were treated with 6 mM salicylate there was also a significant inhibition of the steady-state growth rate during the logarithmic phase; the DT (2.61 hours) was increased by 30-35% with respect to controls. Although the PKA activation shifted the growth recovery of the culture ∼5.22 hours earlier, the slower proliferation phenotype was not suppressed by cAMP addition (Figure 3B). Noteworthy, the growth curve of cells supplied with 6 mM salicylate+cAMP could not overlap with those of cells challenged with cAMP alone because of the different slope. However, they converged at a cell density similar to that used for the initial cell inoculation (that is OD_600_≅0.005) (Figure 3B inset). So, the delay in cell growth recovery caused by 6 mM salicylate appeared to be completely abolished by cAMP, confirming the results seen with 3 mM salicylate and also the specificity of the cAMP suppression effects.

### cAMP signal rescues a strong inhibition of cell growth reactivation and cell lethality caused by salicylate

**Figure 4:**
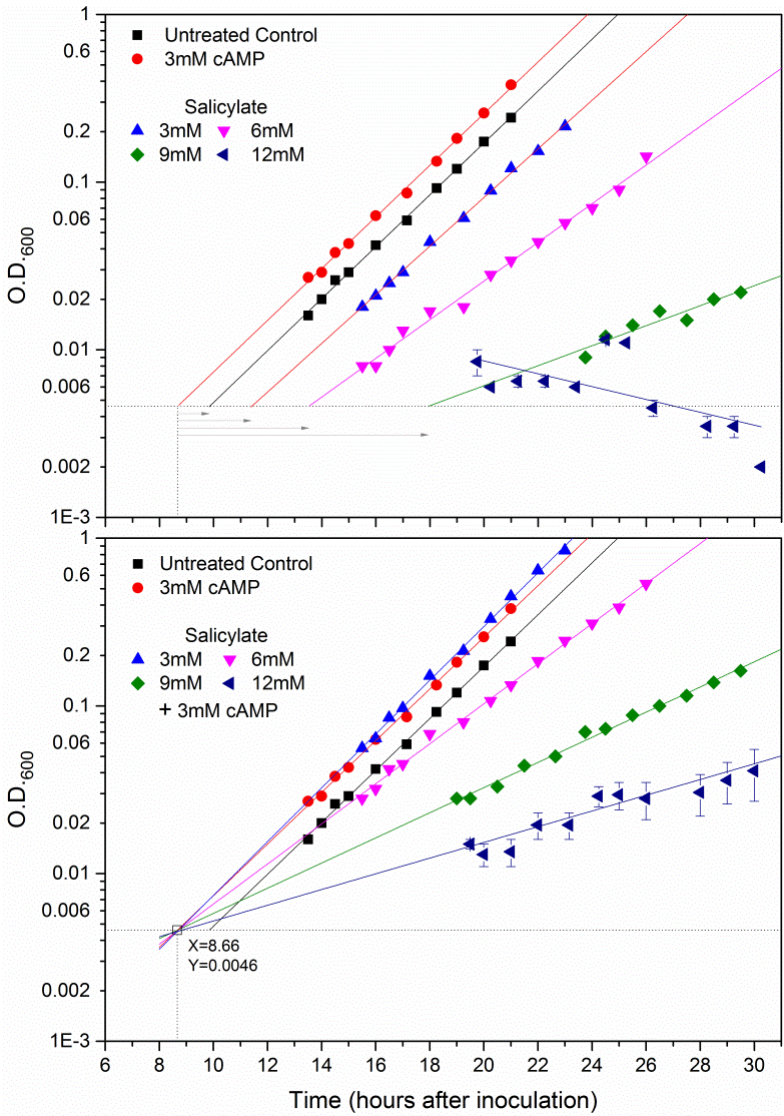
FIGURE 4: Efficient restoration of timely recovery of growth reactivation and rescue of viability of SA-repressed yeast cells by PKA activation. *cyr1*Δ*pde2*Δ*msn2*Δ*msn4*Δ mutant cells were treated with increasing concentrations of salicylate, namely 0 (black ,red; control cells), 3 (blue), 6 (pink), 9 (green)or 12(dark blue) mM SA. The cultures were treated with the increasing concentrations of salicylate in the presence (lower panel) or not (upper panel) of 3mM cyclic AMP (the same symbol for each salicylate dosage is used in both panels). Control cells without salicylate were also supplied (red) or not (black) with 3mM cAMP (upper and lower panel). Growth was monitored as O.D._600nm_. increase and Log_10_ of experimental points were plotted versus time, time zero being when cells were inoculated. The plotted values referring to the conditions of 12mM salicylate plus or minus cAMP are mean O.D._600nm _values ± SD, derived by two time courses followed simultaneously to all the other kinetics. All the experimental points were interpolated by linear regression curves. The values of significant parameters relative to the population growth and viability in these cultures are reported in Tables 1 and 2. There was a progressive inhibition of the exponential growth rate and a systematic increase of the time needed for growth reactivation as a function of salicylate dosage, until proliferation inhibition was a complete at 12mM salicylate (see also main text). In this severe condition the O.D._600nm_ decreased along with time, consistent with a dramatic loss of cell viability within the population (Table 2). The activation of PKAs with exogenous cAMP (lower panel) suppressed the loss of viability and the delay of growth reactivation at any salicylate concentration (see also Table 1). This is graphically shown by a single point of convergence (marked by a clear square) of all the curves with cAMP despite their very different slopes, this point corresponding to the common density of initial inoculums (O.D._600_≅0.005) (for more explanations see main text). Its coordinates (dotted lines) were X=8.66 hours and Y=0.0046 units of O.D._600nm_ as shown in figure. This unique point was considered also the appropriate reference time (see main text) from which the delays of growth recovery due to salicylate could be calculated in the upper panel as represented by the gray right-pointing arrows of increasing lengths (see also Table 1).

A systematic semi-quantitative analysis was performed to compare in a single experiment the growth curves and other parameters of GG104 cells treated with increasing concentrations of salicylate (namely 0, 3, 6, 9 and 12 mM) in the presence or not of cAMP. The results generated a quite consistent picture relative to the antagonism between salicylate and the cAMP signal (Figures 4, S6C, S7 and Tables1, 2; described in detail below ).

**Table 1:** cAMP suppression of salicylate-induced growth reactivation delay. ^a^ The calculated values refers to the experiment with GG104 reported in Figure 4; ^b ^this value refers to the experiment of Supplementary Figure S6B. The salicylate induced delay is calculated by taking either untreated control cells (0mM SA, 0mM cAMP) or the cAMP-treated population as a reference

**TREATMENT^a^**		**Doubling Time (hours)**	**Relative Doubling Time**	**Time of growh recovery (hours)**	**Relative time of growth recovery (hours)**	
Control	---	1.94	set as 1.00	9.84	1.18	set as 0.00
	3.0 mM cAMP	1.95	1.01	8.66	set as 0.00	-1.18
3 mM salicylate	---	2.07	1.07	11.4	2.74	1.56
	0.3 mM cAMP	1.81	0.94	nd	nd	0.05^b^
	3.0 mM cAMP	1.87	0.96	8.7	0.04	-1.14
6 mM salicylate	---	2.61	1.35	13.5	4.84	3.66
	3 mM cAMP	2.52	1.30	8.69	0.03	-1.15
9 mM salicylate	---	5.05	2.60	17.9	9.24	8.06
	3 mM cAMP	4.01	2.07	8.65	- 0.01	-1.19
12 mM salicylate	---	no growth	no growth	>30.5	>21.84	>20.66
	3 mM cAMP	6.42	3.31	8.80	0.14	-1.04

There was a progressive increase of both the time needed for growth reactivation and the mid-log population DT with the salicylate dosage (Figure 4 upper panel and Table 1). Only the first defect was suppressed by cAMP (see below for further details) while the slower exponential growth rate was not corrected by PKA activation, if not very slightly (Figure 4 and Table 1) in agreement with the results presented in previous section. In addition, the highest concentration of salicylate (12 mM) completely inhibited yeast cell growth (for at least 33 hours) (Figures 4 upper panel, Supplementary Figures S6C and S7). In this extreme condition the apparent decrease of the OD_600_ values over time (same figures) prompted us to measure the cell viability within the population (as colony forming unit, CFU). In fact there was a loss of cell viability that became particularly dramatic after 20 hours from inoculation and involved virtually all cells after 40 hours (Table 2). Strikingly, this new phenotype was fully rescued by the activation of PKAs with the exogenous cAMP. In addition, these cells kept alive by an elevated cAMP signal also started to grow, although at a slow rate (DT = 6.4 hours) (Figure 4 lower panel, Tables 1 and 2; Supplementary Figures S6C and S7), reaching remarkable cell densities after 2 days (Supplementary Figure S7, left scale).

It was also evident that all the growth curves of cells stimulated by cAMP strikingly converged at a single point with coordinates Time(x) = 8.66 hours (cells were inoculates at time 0) and OD_600_(y) = 0.0046 (see *Materials and Methods*) irrespective of the presence of salicylate at any of the used concentrations (so even at 12 mM salicylate) (Figure 4 lower panel; see also Supplementary Figure S6C). The cell density of 0.0046 identified with this larger analysis corresponded again to that of inoculated cells (OD_600_≅0.005), so confirming and extending what we preliminarily described in the previous section (Fig.3 inset). We concluded that the specific time in which all these growth curves converge can be considered as the common observable time in which cAMP pushed growth reactivation, in a dominant way with respect to the inhibition due to salicylate (Figure 4 and Table 1; Suppl. Fig. S6C). The growth recovery delay due to each of the different salicylate dosages was calculated with respect to this reference point, namely as the time corresponding an OD_600 _= 0.0046 in each growth curve minus 8.66 hours (arrows in upper panel of Fig 4, Table 1; arrows in Suppl. Fig. S6C). As already mentioned, this delay systematically increased with salicylate concentration until there was a complete growth arrest with 12 mM salicylate accompanied by extensive cell death. As expected the untreated control cells also showed a delayed growth reactivation compared to the cAMP supplied cells (~1.19 hours) (Figure 4 and Table 1).

**Table 2:** cAMP rescue from yeast cell lethality due to salicylate. ^a ^Time spent in fresh medium after the inoculum of GG104 cells; ^b ^Two culture (#1 and #2) were monitored in parallel; ^c ^Viability was calculated as percent respect to CFU of untreated control cells at the inoculum.

**Time (hours)^ a^**		**0**		**21**		**26**		**40**	
**Yeast Cultures ^b^**		#1	#2	#1	#2	#1	#2	#1	#2
**Treatment**		**Viability (% CFU^c^)**							
12 mM salicylate	---	101.7	99.1	29.7	38.9	17.1	18.9	1.3	1.9
	+3 mM cAMP	98.9	105.9	146.8	122.4	228.5	160.7	934.5	200.2

Of note, although PKA activation can very efficiently rescue the massive cellular death in these experiments, it could not suppress the G2/M arrest and loss of viability of salicylate treated cells entering the stationary phase, as described in Supplementary Figure S2 (data not shown) consistent with our previous studies 27.

### The inhibitory effect of salicylate is not linked to early steps of glucose catabolism

Our experiments were performed in glucose media where SA induced both the inhibition of glucose transport and decreased trehalose 6-phosphate (T6P) and UDP-glucose levels 13,14. Glucose is mostly phosphorylated by hexokinases (Hxk1, 2) to glucose-6-phosphate, which then fuels glycolysis. Tps1 activity, essential for growth on glucose, converts glucose-6-phosphate and UDP-glucose to T6P which control glucose flux into glycolysis as an allosteric regulator of hexokinases. In order to understand if these early steps of glucose catabolism are involved in SA effects in our system, we repeated the experiments on galactose-based media in which these steps are absent and the highly conserved Leloir pathway is active 28. In galactose, growth recovery of both wt and *cyr1*Δ cells was also strongly inhibited by salicylate (Supplementary Figures S4A,B and Bonferroni statistical analyses in Figure S4C-right panel; Supplementary Figure S5, further described in *Discussion*). So, the possible inhibition of initial glucose catabolism/signalling by salicylate does not explain the inhibitory phenotypes described here.

## DISCUSSION

Aspirin is one of the most commonly used drugs 1. Adoption of an Aspirin chemoprevention strategy against CRC (colorectal cancer) and other neoplastic diseases (now based on robust evidence 29) will greatly benefit from a better understanding of the mechanisms of action of Aspirin 30. Several cancer prevention properties of Aspirin are mediated by specific activities of salycilate (SA) affecting several cell processes conserved in eukaryotes, such as glucose metabolism 31,32 or apoptosis (e.g. see refs 33,34 and citations therein). So, it is not surprising that some of its established targets in mammals, e.g AMPK 31, GCN2 35, mTORC1 36 and HMGB1^37^, reveal key biological functions from yeast to man.

The budding yeast is a convenient model in which to study how SA can affect processes relevant to neoplastic growth and metabolic disorders 12. Our study provides a phenotypic platform on which SA-mediated mechanisms, which may be conserved in humans, could be further investigated. We have characterized some SA effects on yeast cell growth and survival, in particular during the crucial transitions from or toward quiescence. Cell quiescence has been the focus of intense research efforts as it can be crucial for maintenance and activation of cancer stem(-like) cells (tumour initiating cells), disseminated tumour cells and dormant micrometastases. Indeed, cancer derived mortality predominantly results from fatal outgrowths derived from these dormant elements even after many years. So, targeting of molecular mechanisms underlying quiescence and growth reactivation may allow sensitization of silent cancer cells to ad hoc therapeutic agents 38,39,40.

In our model system salicylate is able to discriminate between active and quiescent cells. The proliferation of exponential phase cells or cells previously arrested in the stationary phase for a short time were almost completely insensitive to salicylate, given even at doses above the maximum serum concentrations associated to Aspirin therapies (Figures 1, S1 and S3). In contrast quiescent yeast cells were extremely sensitive to SA even at low dosages. Indeed, the exit from a profound quiescent state can be strongly delayed in proportion to the drug concentration and, significantly, cells were already inhibited with dosages overlapping the SA blood levels reached during low-dose Aspirin cardioprevention therapies 3,8,9,10 (Figures 2A and S4).

The same dramatic inhibitory response was observed by treating a strain which cannot produce 3’-5’-cyclic-AMP (GG104; significant phenotype: *cyr1*Δ*pde2*Δ*msn2*Δ*msn4*Δ) (Figures 3 and 4 upper panel; Supplementary Figure S6), used here because PKAs can be conveniently activated by exogenous cAMP. In this way we demonstrated that the cAMP signal is a limiting factor for taking the cells out of deep quiescence (Figure 2B), in accordance to its known roles in response to glucose 22. Importantly, this cAMP ability efficiently suppressed the opposite inhibitory action of salicylate delaying or even blocking the growth recovery of G0 cells (Figures 3, 4, S6, S7 and Table 1). We concluded that there is a true antagonistic relationship between the anti-inflammatory and cancer-preventing drug salicylate and key activities of the cAMP signalling pathway in quiescent cells resuming growth.

We tested the suppressive cAMP properties with increasing SA concentrations producing more and more harsh physiological conditions. The duration of the lag time before growth recovery of quiescent GG104 cells progressively increased as a function of SA dosage until, at the highest concentration (12 mM), cell growth was completely inhibited and the cell viability dropped to about 1% with respect to the untreated control cells. There was also a progressive inhibition of the exponential growth rate starting at 6 mM SA (Figures 4A and S6, S7; Table1 and 2). Strikingly, PKA activation very efficiently suppressed both the delayed activation of growth at any SA concentrations and the massive cellular death but was completely unable to increase the growth rate (Figures 3, 4, S6, S7; Tables 1 and 2). This further supports the existence of specific mechanisms affected by the two antagonistic molecules (Supplementary Figure S8A).

Of note, proper entry into G1/G0 phase was also impaired by salicylate (Supplementary Figure S2) and the cell features observed so far suggest that *TOR2 *could be a main target inhibited by SA 41,42. Further analyses on cell cycle and actin cytoskeleton could further support this hypothesis and will be followed by specific molecular analyses.

In order to understand if SA effects involve to some extent the early steps of glucose catabolism in our system, we repeated some key experiments on galactose-based media observing that growth recovery of both wt and *cyr1*Δ cells was strongly inhibited by salicylate (apparently even more than in glucose; see Supplementary Figure S4 and its statistical analyses comparing the response in different media; Supplementary Figure S5). So, the possible impairment of initial glucose catabolism/signalling by salicylate cannot explain the inhibitory phenotypes described here. Interestingly, in the respiro-fermentative regimen driven by galactose the cAMP suppressive activity was reduced in a consistent manner (Supplementary Figure S5), highlighting an important difference in PKA contribution. This is somehow expected as PKA acts positively on cell processes of rapid fermentative growth but negatively on properties associated with slow respirative growth (discussed in 22). In line with our model, in mammals the cAMP/PKA signalling pathway plays an important role in tumour metabolic (glycolytic) adaptation to oxygen insufficiency 43,44,45,46 or to the process of triggering the aerobic glycolysis of cancer associated fibroblasts supplying pyruvate and lactate to tumour cells 47.

Among the possible mechanisms affected by the PKA-SA interaction we will briefly discuss the involvement of AMPK, based on the literature and our preliminary *in silico* analyses on the protein structure and sequence (see *Materials and Methods*). Salicylate is a bona fide activator of AMPK by direct binding to its β1 subunit in higher eukaryotes 31. AMPK is a ubiquitous key energy/metabolic sensor controlling metabolism, cell growth, and survival of eukaryotic cells 48. In *S. cerevisiae*, the basic regulation and function of the AMPK αβγ trimeric structure are conserved (AMPKα is named Snf1). In both yeast and humans, AMPK/Snf1 activation promotes oxidative metabolism used in quiescent cells, rather than the rapid glucose uptake and glycolysis used in many circumstances by proliferating cancer cells and yeast cells cultured in standard 2% glucose media 49,50,51.

In yeast antagonistic phenotypic effects 52 and reciprocal inhibition between Snf1 and cAMP-PKA pathways have been described. Snf1 can negatively regulate PKA by Adenylate Cyclase phosphorylation 53 and Snf1-induced phosphorylation of PKA regulatory subunit Bcy1^54^. A (modest) role in Snf1p inhibition is played by PKA phosphorylation of Snf1-activating kinase Sak1 55. The PKA-dependent dephosphorylating activity of Glc7 PPase on Snf1-Thr210 increases in high glucose 56 inhibiting Snf1/Sip1 localization at the vacuolar membrane 57. Finally, PKA together with DNA checkpoint kinases (Mec1/ATR and Tel1/ATM) inactives Snf1p by Mms21-dependent SUMOylation, as a response to both DNA damage and glucose 58. Thus, if one assumes that yeast AMPK is also activated by salicylate the above mechanisms could contribute to some phenotypes shown here.

It has been known that negative relationships between AMPK and PKA pathways also exist in normal and cancer mammalian cells. PKA can directly phosphorylate AMPK at multiple sites 59,60 among which α1-*Ser485* (cf. 61, but the α1-Ser496 is the actual position, hence it will be used here) is a target for AMPK inhibition in primary hepatocytes, producing resistance to metformin 61. Noteworthy, salicylate can avoid PKA-driven α1-Ser496 phosphorylation so that cAMP effects are prevented in both cultured hepatocytes and *in vivo *61. AMPKα1-Ser496 is located within regulatory Carboxy Terminal Region (CTD), which shows a modest sequence homology but a striking functional and structural (our *in silico *analyses; 62,63) conservation between yeast and humans. However, although α1-Ser496 (^493^RSG**S**VSN^500^) belongs to a conserved CTD sub-region it lacks on Snf1, corresponding to a cluster of three negatively charged amino acids (^603^S**EDE**MST^609^), whose significance is unknown.

In contrast, PKA and AMPK associate and PKA phosphorylates the highly conserved (*in silico *analyses; 62,63) AMPKα1-Ser184 (Snf1-Ser211) 50,51 in primary adipocytes to promote lipolysis, blocking the activating phosphorylation of adjacent conserved key threonine 50,51 of the activation loop (α1-Thr183/Snf1-Thr210) 60. The PKA-driven AMPKα1-Ser184 phosphorylation and the complexes between the proteins were found in different human cancer cell lines, suggesting they can have a broader importance 60. This was strikingly shown by Favre and collaborators that demonstrated how the antitumor effects of AMPK in hepatocellular carcinoma (HCC) cells can be greatly reduced by PKA-driven AMPKα1-Ser184 phosphorylation and the consequent diminution of AMPK activation 64.

Based on our finding and the literature we propose that in *S. cerevisiae *i) one of the three yeast AMPK β subunits could also mediate a direct allosteric Snf1 activation by binding salicylate and ii) at least Snf1-Ser211 might be a key target mediating direct Snf1 repression by PKA. Accordingly, Snf1-Ser211 is phosphorylated *in vivo* and is located within a predicted PKA phosphorylation site (*in silico* analyses; 65,66,67,68). The use of SA-treated strains bearing mutated/null versions of Snf1 (and its subunits), cAMP-pathway genetic manipulations and direct biochemical analyses have been planned to test these hypotheses. Of note, mammalian AMPKα is functional in otherwise lethal *snf1*Δ cells and key elements of its regulatory cascade are also conserved in yeast 69.

AMPK activation is central for cell cycle arrest and apoptosis of glucose-starved HCC characterized by high glycolytic flux 64. So, it is tempting to speculate that PKA hyperactivation could prevent AMPK/Snf1 induced growth inhibition and even cell lethality in both glucose-starved yeast cells and some cancer cell types with similar mechanisms responding to salicylate.

In general, deregulation of PKA has been related to both initiation and progression of cancer, and has been observed in different types of human cancer and cancer-associated stromal cells (43,44,45,46,47 and citations therein). Salicylate might act as an anti-cancer drug opposing to some activities of the cAMP-PKA signalling pathway. Perhaps more importantly, our work further emphasize that fundamental cell choices are linked to a fragile balance based on metabolic controls, subverted in many ways in tumour cells (Supplementary Figure S8B). This view is strongly strengthened by the fact that salicylate can interacts with some important metabolic hubs conserved in eukaryotes (as indicated by the roles of its established targets in vertebrates; see above), as well as by a recent demonstration that aspirin/salicylate behaves as a bona fide caloric restriction mimetic 70 showing a pro-autophagic activity dependent on direct SA inhibition of EP300 acetyltransferase activity (with a conserved mechanism) 71. Intriguingly, EP300 also mediates cAMP driven gene regulation by binding to CREB protein 72. The observation that starved SA-treated yeast cells have an altered control of last cell divisions not responding to PKA activation (Supplementary Figure S2) supports an even more complex picture of salicylate pleiotropic activities.

As shown in a model summarizing our findings (Supplementary Figure S8), the budding yeast *S. cerevisiae *can provide an unique opportunity to dissect complex conserved mechanisms whose instrumental modification sustain cancer, and to better design their pharmacological control. For instance, it may be desirable to find salicylate derivatives that are more specific for one of its targets or that are no longer antagonized by specific proliferative or anti-apoptotic signals important in certain circumstances (e.g. when they control the metabolism), such as in the case of cAMP. We wonder if the dramatic responses of long-lasting quiescent yeast cells described here could be somehow related to the mechanisms of growth reactivation and survival of dormant cancer cells. This idea, if proven, would greatly increase the importance of our model for identifying new molecular targets/strategies of anti-cancer therapies.

## MATERIALS AND METHODS

### Yeast strains

The yeast strains used throughout this work (when not otherwise specified) were W303-1A (*MATa leu2-3,112 trp1-1 can1-100 ura3-1 ade2-1 his3-11,15*) 23 and its derivative GG104 (*cyr1::kanMX pde2::TRP1 msn2::HIS3 msn4::TRP1 leu2-3,112 trp1-1 can1-100 ura3-1 ade2-1 his3-11,15*)^24^. Other strains used were SP1 (MATa his3 leu2 ura3 trp1 ade8 can^r^) 25 and a fully prototroph version of W303-1A (WP, prepared in our laboratory).

### Media and growth conditions

Yeast cells were cultured at 30°C in SD medium (Yeast nitrogen base without amino acids and with ammonium sulphate 6.7 g/l, Glucose 2%, supplemented with Adenine sulphate 60 μg/ml, Uracil 20 μg/ml, l-Tryptophan 40 μg/ml, l-Histidine hydrochloride 20 μg/ml and/or l-Leucine 60 μg/ml whenever needed by the presence of the corresponding auxotrophies) or the proper selective drop-out CSM (Complete Synthetic Medium, supplied by ForMedium, United Kingdom). When required, glucose was substituted with 2% galactose. All the experiments were performed at 30°C in warm room or into an orbital-shaker incubator (only for experiments of Figure 1B). Three independent repeats (defined as measurements taken during different experimental runs) of each experiments were performed (with the exception of the two repetitions of the preliminary analyses shown in Supplementary Figure S5). Repeats of several experiments were performed in triplicate (as specified in figure legends), duplicate (Figs. S1C and S7) or as a single series of measurements (when it was needed to favor the simultaneous analysis of a large number of treatments whose effect had already been explored individually (e.g. the overall experiment of Fig.4; but even in that case, however, the treatments previously showing the most extreme responses were followed in duplicate). All results showed evident consistency and it was always possible to reach the same key conclusions on the biological effects through all similar analyses. Statistics was used for necessarily more quantitative comparisons (Supplementary Figure S4). Aged cell cultures were obtained by incubating cells at 30°C in their original medium from 1 day to 3 weeks. An equimolar 0.5 M stock solution of Salicylic acid and Sodium Bicarbonate (Sigma, S5922 and S5771, respectively) was freshly prepared every week. This solution was diluted at the different salicylate concentrations into the growth medium. cAMP (Sigma, A9501) was weighed and added directly to the medium.

### Cell parameter measurements

Growth of cultures as indicated by an increase in cell density (≅biomass) was monitored as Optical Density at 600 nm (OD_600_) with a Amersham Pharmacia Ultrospec 1100 Pro Spectrophotometer (Amersham Pharmacia Biotech Ltd, UK). Growth in terms of increase in cell number was followed by using a Coulter Counter model Z2 (Coulter Electronics, Inc.). Cell size analyses were performed using a Coulter Z2 Particle Cell Analyzer (Beckman-Coulter) coupled to the Coulter Counter, obtaining cell volume distributions of yeast populations. The fraction of budded cells was scored by direct microscopic observation on at least 400 cells, fixed in a buffered (pH 7.2) solution of 2.6% formaldehyde and mildly sonicated. Cell viability was assessed by cell plating on Yeast-peptone-destrose solid medium (YPDAT; 2% Agar, 2% glucose, 1% yeast extract and 2% bacto peptone plus Adenine and Tryptophan) through colony forming unit (CFU) counting. Contrast phase light microscopy photographs of yeast cells were obtained with an Olympus CH20 Microscope (Olympus Corp.).

### Flow cytofluorimetric analysis

A total of about 2x10^7^ cells for each sample were collected by centrifugation, fixed in 70% ethanol, stored at 4°C and subsequently processed for flow-cytometry. DNA staining was performed essentially as described previously with some modifications 73. Cells were washed once with PBS, resuspended in 1 ml of PBS with RNAse 2 mg/ml and incubated for 4 h and 30 min at 37°C. After incubation, cells were washed once with PBS, resuspended in DNA staining solution (Propidium Iodide 0.046 mM, Tris 0.05 M, MgCl_2_ 15 mM pH 7.5) and incubated in ice and dark for about 45 min. All the washing and staining steps were performed in 1.5 ml Eppendorf tubes and centrifugations at 16000 x g for 2 min at 4°C. Cell suspensions were transferred in FACS Falcon tubes and sonicated twice for 10 s before analysis. Analyses were performed using a CYTOFLEX S Beckman Coulter equipped with a 488-nm laser emission. Plot generation was performed with CytExpert 2.0 software (Beckman Coulter).

### Graphics and statistics

All graphics processing and calculations as well as growth curve fittings and statistics were performed using OriginPro9.1 (64bit), a data analysis and graphing software (OriginLab Corporation, One Roundhouse Plaza, Suite 303, Northampton, MA 01060, USA).

### *In silico* analyses

The 3D structure and sequence of AMPKα (in particular its CTD) were compared between yeast and humans protein version using PyMOL 62 and PROMALS3D 63 programs. The conservation of Snf1-Ser211 was confirmed by comparing the sequence of AMPKα among a large number of species.

The putative PKA phosphorylation sites were searched with pkaPS on line program 65,66. The phosphorylated residues of AMPK and Snf1 (with the associated consensus sequence for different protein kinases) both *in vivo* and *in vitro *were scored using the PhosphoSitePlus® (PSP) and/or PhosphoGRID databases 67,68.

This work was supported by Scientific Research DOR funds (2016-2017) from the University of Padua to MDB and FAR 2016 grant from the University of Milano-Bicocca to EM.

## SUPPLEMENTAL MATERIAL

Click here for supplemental data file.

All supplemental data for this article are also available online at http://microbialcell.com/researcharticles/antagonism-between-salicylate-and-the-camp-signal-controls-yeast-cell-survival-and-growth-recovery-from-quiescence/.
