# Supplementary material for: Antagonism between salicylate and the cAMP signal controls yeast cell survival and growth recovery from quiescence
Source: Microb Cell. 2018 Mar 26;5(7):344–56. doi: 10.15698/mic2018.07.640 (PMC6035838; doi:10.15698/mic2018.07.640)
Supplement: Supplementary file 1 [file mic-05-344-s01.pdf]

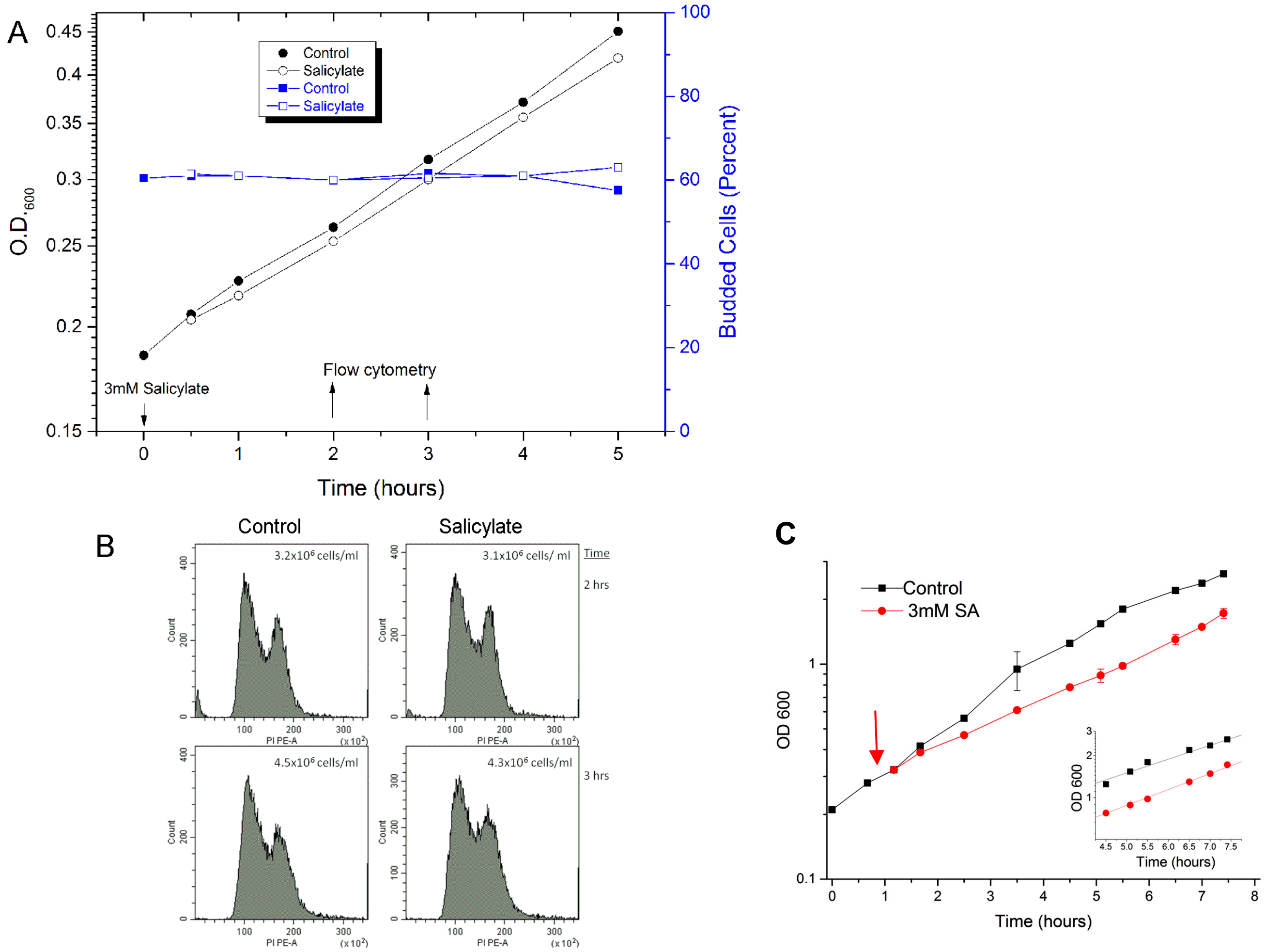

Supplementary Figure S1. Unaltered Cell cycle parameters during steady state exponential growth of SA-treated yeast cells. This kind of experiment was described in Figure 1 legend. The percent of budded cells **(A)** and the DNA content distributions **(B)** of SP1 strain grown in SD medium were obtained at the times marked in figure, during mid-log growth phase. The same experiment was performed in duplicate with W303 grown in CSM **(C)** being the plotted points mean values (± SD), derived by the two parallel time courses (each ± salicylate; arrow marks the SA addition). Last six points have been interpolated (linear regression) to better show that the growth curves returned steadily parallel to that of the control after 2-3 hours from SA treatment (**inset**), as shown in panel A and Figure 1. Symbols: (●)SA treated cultures, (■) control cells.

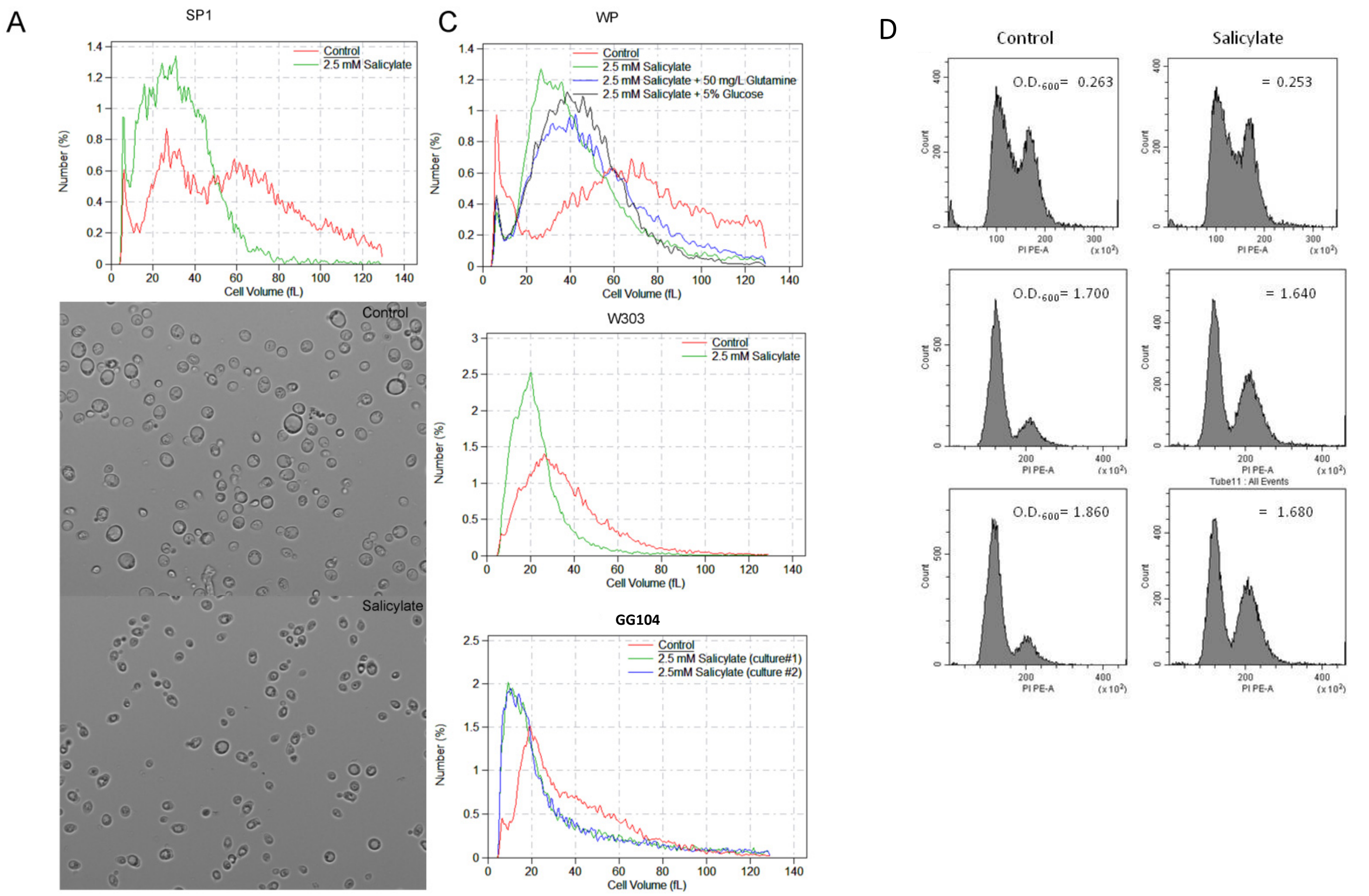

B

Effect of salicylate on growth parameters of stationary phase yeast cells

3mM Salicylate	-	+	-	+	-	+	-	+
Strain	Cells per ml		O.D.$_{600}$		Mean Cell Volume(fL)		% Budded Cells	
SP1	$1.06x10^8$ 53.5%*	$5.58x10^7$ 2.2%*	4.29	1.96	56.8	32.2	23.6	54.2
W303-1A	$1.09x10^8$	$8.62x10^7$	3.58	1.90	36.6	23.4	28.5	53.8
W303 prototroph (WP)	$2.26x10^8$	$1.63x10^8$	4.65	2.30	64.7	43.3	20.0	56.1
WP+ 2%Galactose	nd	nd	3.74	1.91	nd	nd	6.5	25.0
WP+5%Glucose	nd	$1.15x10^8$	nd	2.30	nd	44.4	nd	
WP+50mg/L Glutamine	nd	$1.69x10^8$	nd	2.00	nd	49.7	nd	

*Viability, calculated as CFU per cell number (determined by Coulter Counter counts)

E

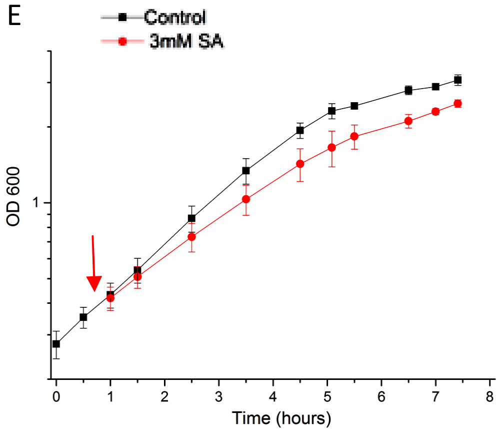

Supplementary Figure S2. Defective arrest in G1-unbudded phase of yeast cells in the presence of salicylate. (A) Cell volume distributions and microphotographs of SP1 *wt* strain treated (green line) or not (red line) with salicylate, after 12 hours into stationary phase. **(B)** Table reports significant growth and cell cycle parameters and viability of SP1 and other strains in stationary phase, as described for panels A and C. **(C)** Cell volume distributions of WP, W303 and GG104 cells in stationary phase ± SA. WP strain was grown also in different nutritional conditions (Table in B and uppermost panel). **(D)** DNA content distribution of logarithmic (uppermost panels) or stationary phase (other panels) cells ±SA. O.D. values are reported as a reference to the growth phase (in minimal medium). **(E)** W303 strain cells treated (●) or not (■) with SA were followed until they were approaching the stationary phase. The means ±SD represent one experiment performed in triplicate.(Arrow: SA addition). Note the lower density reached by the SA-treated culture.

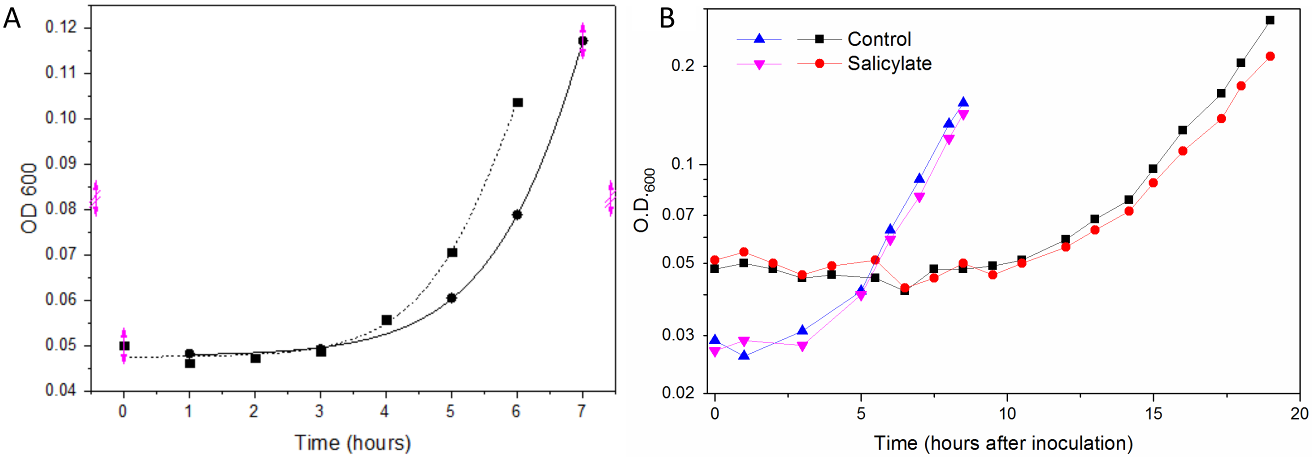

Supplementary Figure S3. The time of growth recovery of chronologically young yeast cells was not affected by Salicylate whereas it increased with the time spent into G0 phase. (A) *Lag Time* increase as a function of the time previously spent into stationary phase. W303 yeast cells were re-inoculated into complete synthetic medium containing all 20 amino acids (100µg/ml; Leucine 200µg/ml), Uracil (100µg/ml), Adenine (100µg/ml) and Inositol (200µM) after 2 (■) or 4 days (●) spent into stationary phase. The OD600 values of the graph are the mean values (without SEM) of control cells reported in Figure 1B (upper and lower part). Data were interpolated with a non-linear fit and the inoculum density of the "4-days" curve was normalized to that of the other curve for a more direct comparison. *Lag Time* difference was ~40 minutes. **(B)** Unaltered *lag time* before growth recovery of chronologically young yeast populations treated with salicylate. W303 yeast cells were re-inoculated into fresh minimal synthetic medium after 17hrs (▲,▼) or 4 days (■,●) spent into stationary phase. O.D.$_{600nm}$ values were monitored in control untreated cells (▲,■) and cells supplied with 3mM salicylate (▼,●) and their Log$_{10}$ values plotted against time. Doubling times during the early log phase were: 1.91 (▲),1.92 (▼), 2.69(■) and 3.09(●) hours. Nutritional conditions were more stringent and the *lag time* differences between cells previously left for different times in G0 were more evident than in (A), but the time of growth recovery was still unaltered by salicylate (see also Figure 1B).

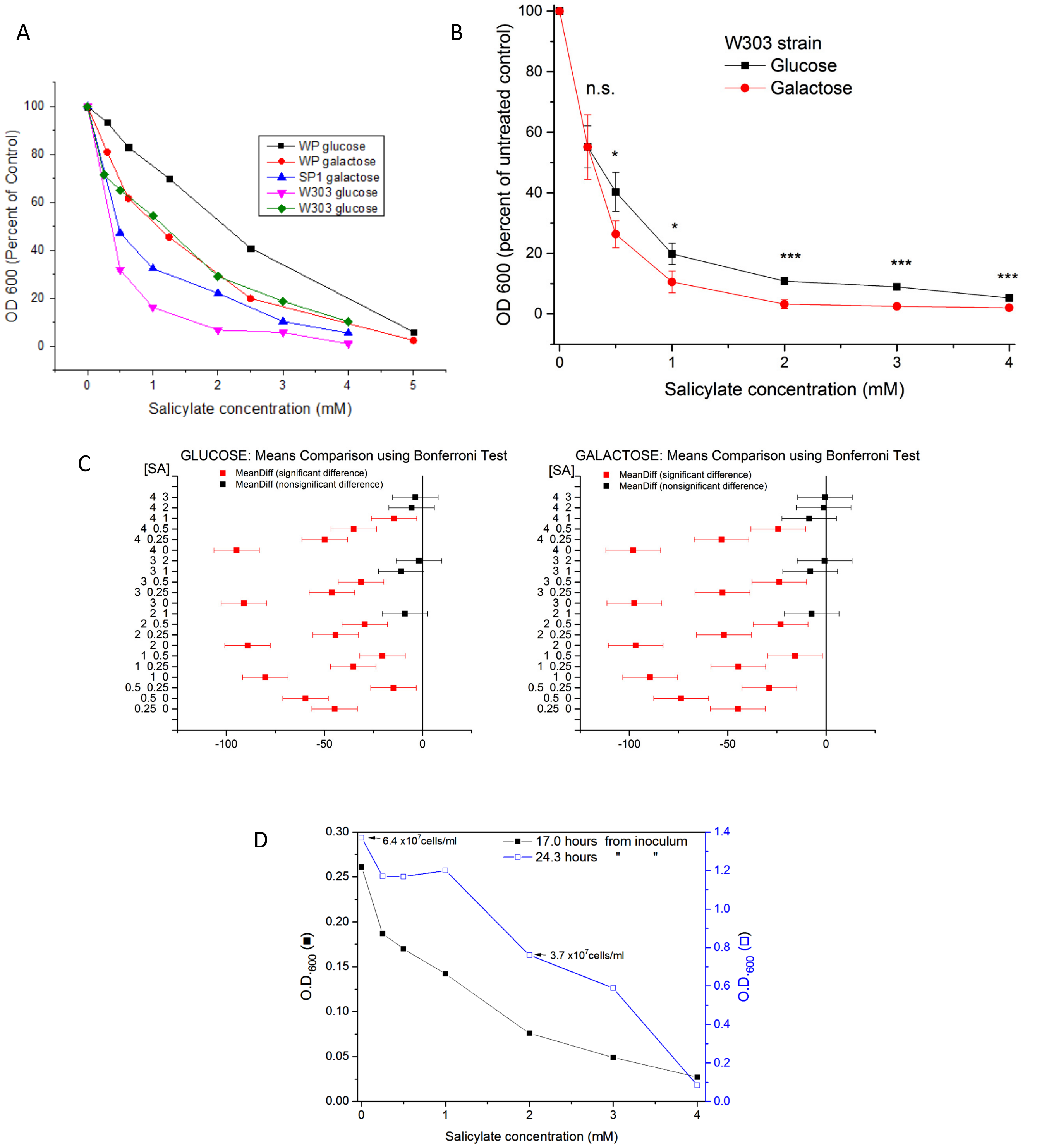

Supplementary Figure S4. Growth recovery of long-term stationary phase cells is strongly delayed by salicylate treatment in both glucose and galactose based media. **(A)** The experiments were performed as described in Figure 2A legend except that different strains and carbon sources were used. Results were compared after normalizing the O.D. values on those of control (no salicylate). The absolute $O.D._{600}$ measurements were between 0.2 and 0.4 units in each SA-free medium. The remarkable response variability that can be observed is in part due to the great sensitivity of this assay to changes in physiological conditions (see also the time course in panel D). **(B)** The same experiment were done in triplicate both in glucose and galactose in order to perform statistical analyses. In each medium and at any SA dosage there was a significant SA-induced growth inhibition respect to the untreated controls (2ways-ANOVA analyses; not shown). Difference between glucose and galactose were significant or highly

significant at each dosage with the exception of 0.25mM SA (as specified by asterisks in figure; t-Test analyses). **(C)** Differences among various SA dosages within the experiment with each C-source were in most cases significant (Bonferroni comparison). The compared salicylate doses ([SA]=mM salicylate concentrations) are written in pairs to the left of the graph. **(D)** Reversibility of salicylate effects. Cells of the experiments reported in Figure 2A were monitored again after 24.3 hours (□). Continuous growth in the presence of salicylate indicated that growth inhibition due to the drug was reversible. Cell numbers were roughly proportional to optical densities, as exemplified in the graph.

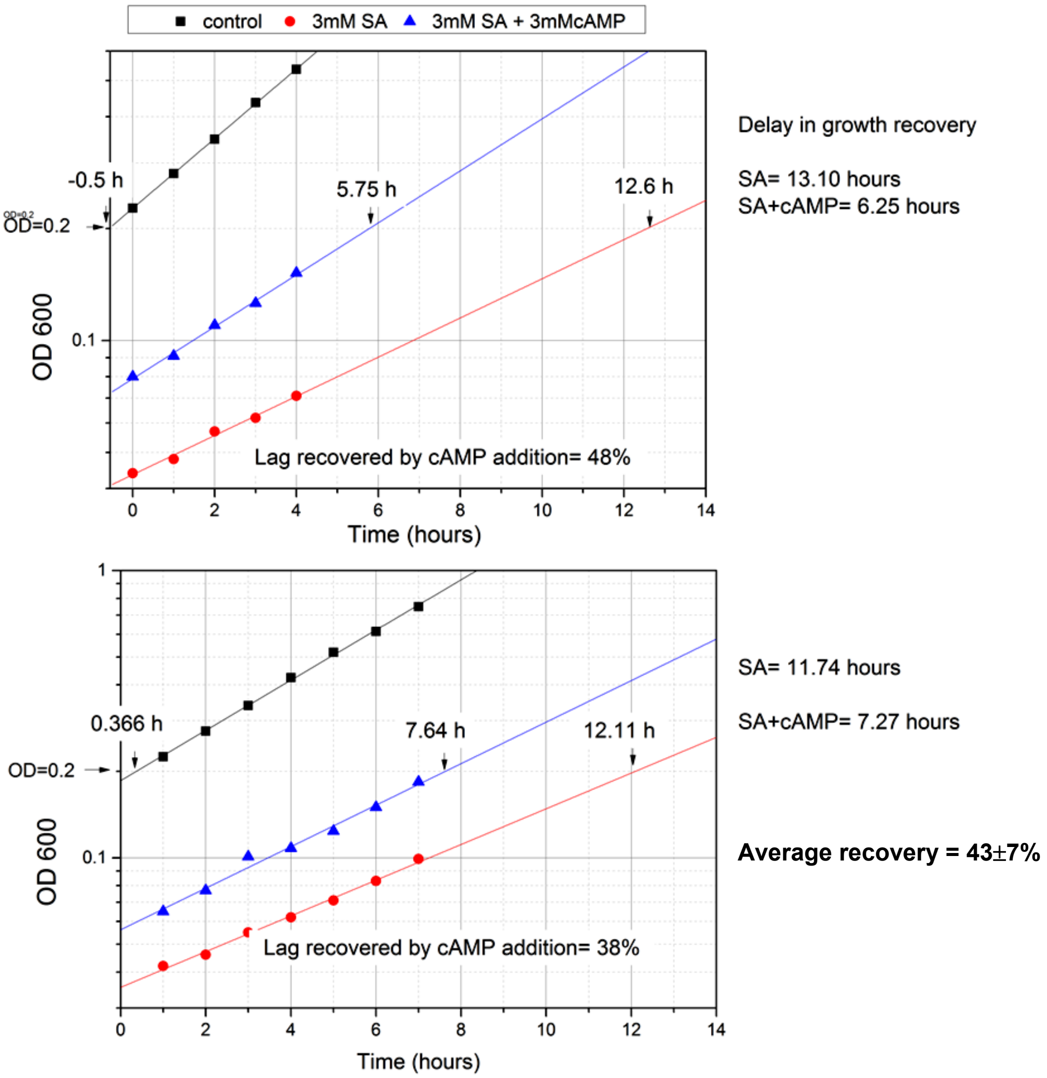

Supplementary Figure S5. cAMP-driven suppression of SA-induced delay of growth recovery in galactose. The two independent experiment repeats with GG104 strain shown in figure were performed as described in Figure 3A legend except that glucose was replaced by galactose. The delay of growth recovery was calculated taking as reference an OD600=0.2 units (arrows) (see main text for explanations). In galactose the percent of recovery allowed by cAMP was 43±7% (calculated as Mean±SD of the two experiments). The same parameter was much higher when cells were growing on a fully fermentable carbon source (glucose), being 175.7±19.5% (mean of three experiments; range:156%-195%, values calculated from experiments of Figs.3A,B and S6A)

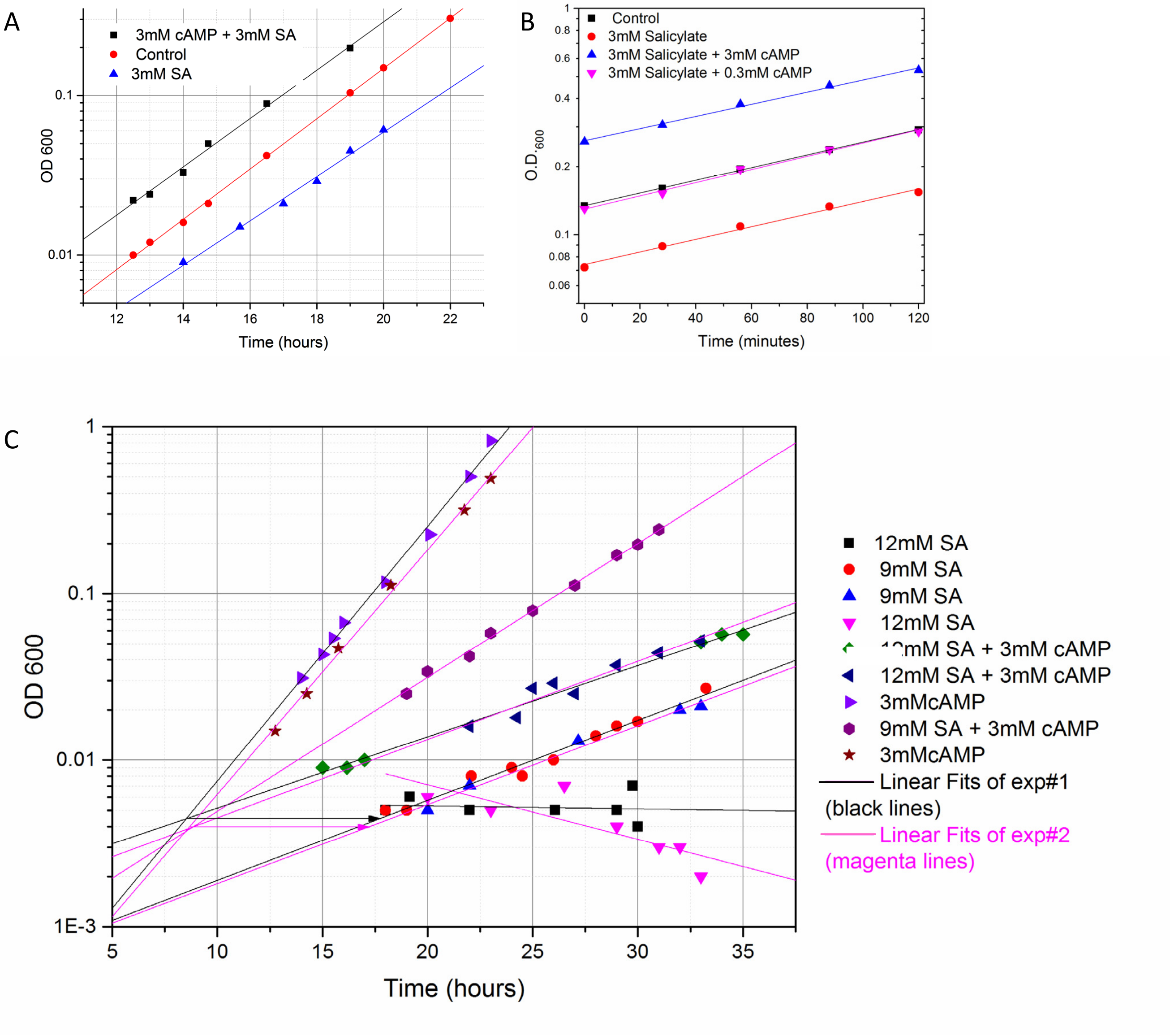

Supplementary Figure S6. Independent repeats of experiments presented in main Figures 3 and 4. **(A)** This experiment was used together with those in Panel B and Figure 3A to calculate the percent of recovery allowed by PKA activation reported in Fig.S5 legend. Noteworthy, The curve generated by treating cells with cAMP roughly overlapped that with 3mM SA+3mM cAMP (not shown). **(B)** Dose dependency of cAMP effects. *cyr1Δpde2Δmsn2Δmsn4Δ* cells were treated (●) or not (■) with 3mM salicylate, as described in Figure 3. Two salicylate-treated cultures of the same mutant strain were supplied with cAMP at a concentration of 0.3 (▼) or 3.0 mM (▲). The Log_{10} of O.D.$_{600nm}$ values during exponential growth were plotted against time (minutes) and fitted with linear regression curves. The effect of cAMP is roughly proportional to its concentration. In any used condition the exponential growth rate was not modified (generation time ~2.0 hours). The curve generated by treating cells with 3mM cAMP only overlapped that with SA+cAMP(▲), with negligible deviations (not shown) **(C)** The suppressive dominant effects of exogenous cAMP on both the delay of growth recovery and the loss of viability of cells due to very high SA dosages, as shown by two independent experiments. Detailed explanations can be found in main text. Arrows indicate growth activation delay of cells challenged with 9mM SA. In experiment #1 the analysis of cells treated with 9mM SA+3mM cAMP were based only on a few initial points during growth whose fitting was fully consistent with the results of the same treatment in experiment #2 (data not shown). During experiment #2 the viability of cells treated with 12mM SA dropped to 2.7% after 31 hours (not shown).

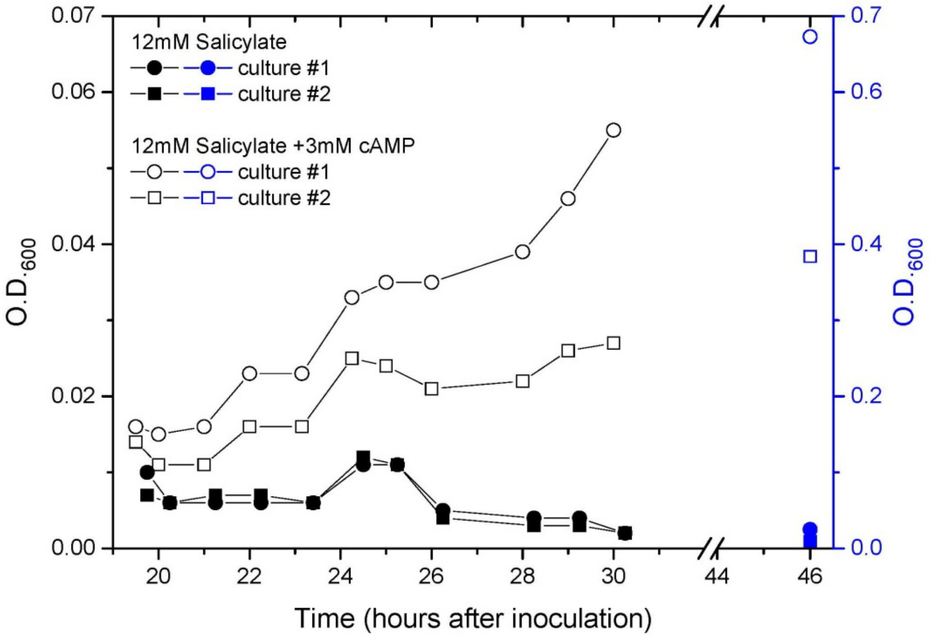

Supplementary Figure S7. Cells exposed to a high salicylate dosage are effectively rescued by PKA activation and show robust growth. Cells (GG104 strain) of two parallel cultures, namely #1 (●,●) and #2 (■,■), supplied with 12mM salicylate underwent an extensive (>99%) loss of viability (see Table 2) that could be rescued by the addition of 3mM cAMP (open symbols: ○,○ for #1 and □,□ for #2). Population growth was followed as O.D.$_{600nm}$ increase for 2 days. The cAMP addition was always able to produce a full rescue although in this extreme condition there was some variability of the cell growth rate among different cultures supplied with cAMP (see also Fig.S6B), as put more in evidence here by linear-scale graphical representation. During six different experiments the worse and best generation times obtained with cAMP were ~7.15 and ~4.40 hours, respectively. In any cases growth was robust and lasted till the cultures reached high cell densities, as can be seen here (namely 0.4 and 0.7 units of O.D.$_{600nm}$) (open blue symbols). Time 0 corresponds to the inoculation time. The 2nd scale on the right is used to plot the terminal OD$_{600}$ after 46.5 hours

A

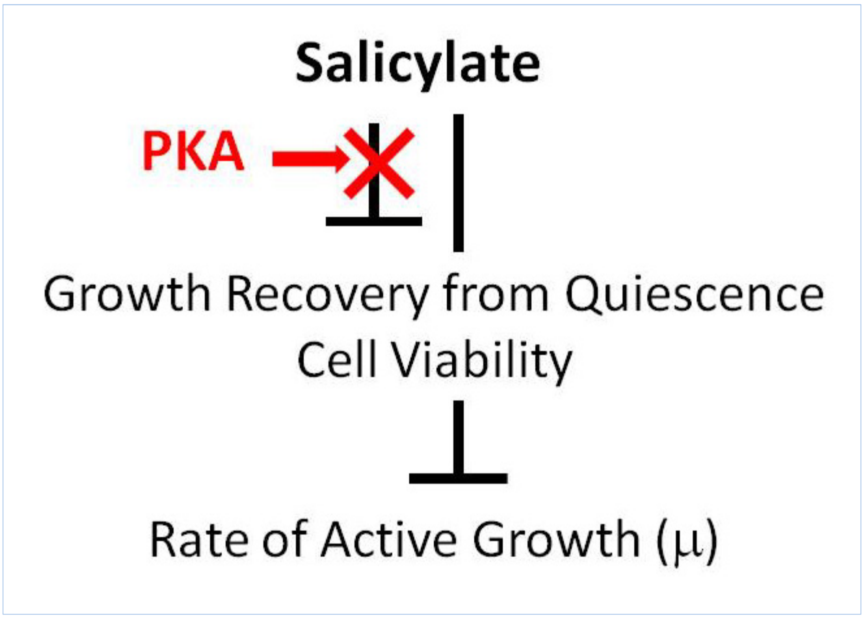

B

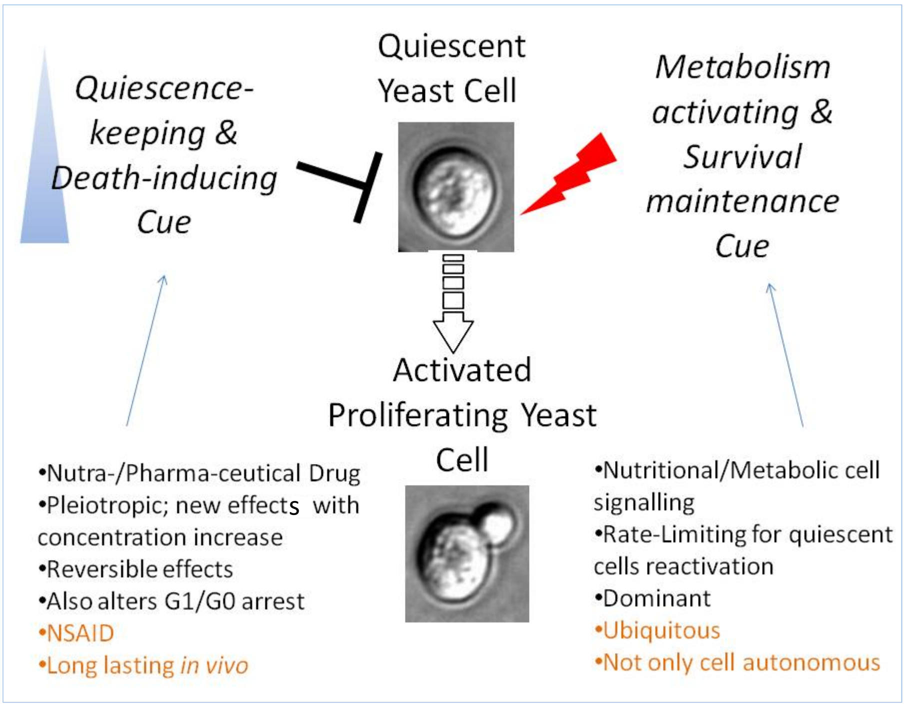

Supplementary Figure S8. A model based on our phenotypic analyes. (A) Summary figure describing the antagonistic effects of salicylate and cAMP signal on important and conserved cellular processes, also relevant for the physiology of dormant cancer cells (see main text). **(B)** A possible more general model that highlights the powerful role of a cue activating metabolism in appropriate nutritional conditions and suppressing cellular quiescence or even cell death controlled by another cue. In the model salicylate may be considered either a tool to highlight the phenomenon or a therapeutic drug. *In vivo*, cell niche conditions and immunity are crucial to control quiescence maintenance and/or cell death of cells capable of initiating or disseminating cancer [74]. Importantly, a right balance between quiescence maintenance and proliferation ability can be the fatal condition leading to highly aggressive relapses from dormant tumours and also characterizing some mechanisms of cancer penetrance [38-40]. Some features of the cues considered into the model are written in black if they are derived from our analyses. More general features are in colour.
